# Anti-inflammatory effects of a casein hydrolysate and its peptide-enriched fractions on TNF*α-*challenged Caco-2 cells and LPS-challenged porcine colonic explants

**DOI:** 10.1002/fsn3.153

**Published:** 2014-09-05

**Authors:** Anindya Mukhopadhya, Nessa Noronha, Bojlul Bahar, Marion T Ryan, Brian A Murray, Phil M Kelly, Ian B O'Loughlin, John V O'Doherty, Torres Sweeney

**Affiliations:** 1 School of Veterinary Medicine, UCDDublin, Ireland; 2 Food for Health Ireland (FHI), UCDBelfield, Dublin, Ireland; 3 School of Agriculture & Food Science, UCDDublin, Ireland; 4 Teagasc Food Research Centre MooreparkFermoy Co. Cork, Ireland

**Keywords:** Bioactive, casein hydrolysate, inflammation, intestine

## Abstract

Bioactive milk peptides are reported to illicit a range of physiological benefits and have been proposed as potential functional food ingredients. The objective of this study was to characterize the anti-inflammatory properties of sodium caseinate (NaCAS), its enzyme hydrolysate (EH) and peptide-enriched fractions (5 kDa retentate [R], 1 kDaR and 1 kDa permeate [P]), both in vitro using a Caco-2 cell line, and also ex vivo using a porcine colonic tissue explant system. Caco-2 cells were stimulated with tumour necrosis factor alpha (TNF*α*) and co-treated with casein hydrolysates for 24 h. Following this, interleukin (IL)-8 concentrations in the supernatant were measured using enzyme-linked immunosorbent assay. Porcine colonic tissue was stimulated with lipopolysaccharide and co-treated with casein hydrolysates for 3 h. The expression of a panel of inflammatory cytokines was measured using qPCR. While dexamethasone reduced the IL-8 concentration by 41.6%, the 1 kDaR and 1 kDaP fractions reduced IL-8 by 68.7% and 66.1%, respectively, relative to TNF*α-*stimulated Caco-2 cells (*P < *0.05). In the ex vivo system, only the 1 kDaR fraction elicited a decrease in*IL1-α*,*IL1-β*,*IL-8*,*TGF-β* and*IL-10* expression (*P *< 0.05). This study provides evidence that the bioactive peptides present in the 1 kDaR fraction of the NaCAS hydrolysate possess anti-inflammatory properties in vitro and ex vivo. Further in vivo analysis of the anti-inflammatory properties of the 1 kDaR is proposed.

## Introduction

Milk is an emulsion of milk fat globules within a water-based fluid of dissolved carbohydrates, minerals and suspended protein particles. While whole milk is a valuable source of nutrition (Mills et al. 2011), there is also a high incidence of allergic symptoms and lactose intolerance in individuals (Haug et al. 2007). The digestion/hydrolysis of milk proteins can yield physiologically important bioactive peptides that have a wide range of biological activities which have been researched extensively in an effort to isolate bioactive ingredients suitable for functional foods (Fox 2001; Madureira et al. 2010; Abete et al. 2011; Phelan and Kerins 2011; Jakubowicz and Froy 2013). These bioactive peptides can be released in vivo by digestion in the gastrointestinal tract or in vitro by fermentation or enzymatic hydrolysis from parent milk proteins (Phelan and Kerins 2011). Casein is a major protein in cow's milk and constitutes about 80% of the total milk protein. Milk bioactives generated from either sodium caseinate (NaCAS) or whey protein are of interest because they are low in lactose and thus have potential as an alternative milk source for lactose intolerant individuals (Sindayikengera 2006).

Several studies have used in vitro gastrointestinal cell models, such as human colonic adenocarcinoma cells (Caco-2 cells) to evaluate the bioactivity of milk hydrolysates: whey protein isolates were identified with anti-inflammatory effects in Caco-2 cells stimulated with hydrogen peroxide (Piccolomini et al. 2012); also a 3-kDa fermentate of NaCAS, generated from the fermentation of lactic acid bacteria, activated the transcription factor NF*κ*B in Caco-2 cells (Stuknyte et al. 2011). Although Caco-2 cells functionally resemble colonic enterocytes, this monolayer of cells lacks immune cells in the basolateral side of the epithelium. Thus, while Caco-2 cells are a useful model of the gastro-intestinal tract (GIT), this model lacks the cellular heterogeneity, present in vivo. Hence, intestinal tissue explants are also used to model the GIT to further validate the in vitro outcomes (Bahar et al. 2012; Girard-Misguich et al. 2012) and to provide an alternative insight into the effects of bioactive compounds on signaling factors and cytokines active within the entire tissue and all the cell types within it (Randall et al. 2011). The RNA present in porcine colonic explants remains stable for 3 h following an lipopolysaccharide (LPS) challenge (Bahar et al. 2012). Hence, this colonic ex vivo system can be applied to evaluate the effects of bioactive compounds on inflammatory cytokine gene expression (Bahar et al. 2012).

A number of cytokines from distinct T-cell subsets (Th1, Th2, Th17 and Treg) plays a central role in intestinal immunology (Roberts-Thomson et al. 2011). Imbalances in these T-cell subsets and their cytokines are central to the etiology of chronic inflammatory bowel diseases (IBDs), including Crohn's and colitis (Powrie 2012) where either inadequate or prolonged activation of the immune system results in chronic mucosal inflammation (Rogler and Andus 1998). Plasma interleukin (*IL*)*-8* has been shown to correlate with intestinal inflammation, establishing it as an important marker of inflammation (Scherl and Longman 2012). Tumor necrosis factor alpha (TNF*α*) is also implicated as a key player in the progression of IBD (Rogler and Andus 1998). Chronic inflammatory disorders, such as IBD, which are generally treated with steroids and immunosuppressants, can have undesirable side effects, considerable toxicity and variable outcomes with respect to controlling symptoms (Rogler 2010). Therefore, natural and safer alternatives are continuously being sought as a means of alleviating IBD symptoms (Khan et al. 2012).

A number of NaCAS hydrolysate fractions with distinct physico-chemical properties have recently been generated in our group. The overall objective of this study was to determine if these fractions have anti-inflammatory biological activity in vitro and ex vivo. Hence, the first objective of this study was to characterize the anti-inflammatory effects of NaCAS and a NaCAS enzyme hydrolysate (EH) along with its associated 5 kDa retentate (5 kDaR), 1 kDa retentate (1 kDaR) and 1 kDa permeate (1 kDaP) fractions in an Caco-2 cell line stimulated with TNF*α*. The second objective of this study was to characterize the anti-inflammatory effects of the same NaCAS and its fractions in ex vivo LPS-challenged porcine colonic tissues.

## Materials and Methods

### Generation of a NaCAS hydrolysate

NaCAS (90% w/w protein, Kerry Food Ingredients, Listowel, Ireland) from bovine milk was suspended at 10% (w/w) on a protein basis in distilled water and dispersed under agitation at 50°C for 1 h using an overhead stirrer (Heidolph RZR 1, Schwabach, Germany). The pH was adjusted to 7.0 using a NaOH 4.0 N solution (VWR, Dublin, Ireland). A bacterial food-grade enzyme preparation was added to the protein solution and hydrolysis was carried out at constant pH (7.0) by manual titration of 4.0 N NaOH, until the desired degree of hydrolysis (DH) was achieved. The enzyme was inactivated by heat treatment of the hydrolysate sample at 85°C for 25 sec. All hydrolysis experiments were conducted in triplicate. The hydrolysate (50 L) described above was dehydrated in a pilot scale Anhydro Lab 3 spray drier (SPX Flow Technology A/S, Soeborg, Denmark) at an inlet temperature range of 185–190°C and outlet of 85–90°C. The EH was further concentrated (to ca 40% total solids) before spray drying, as outlined above, in a Anhydro F1 Lab single-effect falling film evaporator (SPX Flow Technology).

### Membrane processing of the casein hydrolysate

The milk hydrolysate was subjected to microfiltration (MF) using a GEA Model*F* unit (GEA Process Engineering A/S, Skanderborg, Denmark). This unit was fitted with three ceramic membranes (Tami Industries, Nyons Cedex, France) having a nominal molecular weight cut off of 0.14 *μ*m. MF was carried out at 50°C and pH 7 to a volume concentration factor (VCF) of 8. A feed recirculation rate of 1500 L h^−1^ at 1 bar and a membrane inlet pressure of 4.2 bar were maintained throughout processing. The permeate stream prepared above was then subjected to ultrafiltration (UF) using the same GEA model*F* unit fitted with two spiral wound membranes (Koch Membrane Systems, Wilmington, MA). These membranes have a nominal molecular weight cut off of 5 kDa. The 5 kDa permeate stream was finally processed on the GEA model*F* plant fitted with two spiral wound membranes (Alpha Laval AB, Lund, Sweden). These membranes have a nominal molecular weight cut off of 1 kDa. UF was carried out at 50°C and pH 7 to a VCF of 7. A feed recirculation rate of 1500 L h-1 at 1 bar and membrane inlet pressure of 5 bar were maintained throughout processing. The 0.14 *μ*m and 5 kDaR streams described above were dehydrated in a pilot scale Anhydro Lab 3 spray drier (SPX Flow Technology A/S) at an inlet temperature range of 185–190°C and outlet of 85–90°C. The 1 kDaR and 1 kDaP were further concentrated (to ca 40% total solids) before spray drying, as outlined above, in a Anhydro F1 Lab single-effect falling film evaporator (SPX Flow Technology A/S). Thus, at the end of the process a 5 kDaR, 1 kDaR, and 1 kDaP were generated.

### Compositional analysis

The lipid content of the powder samples was determined using the Röse–Gottlieb method for lipid determination (Vester 1962). Ash was determined gravimetrically through modification of the International Dairy Federation (2008) method where >1 g of powder was weighed to the nearest 0.1 mg. Dry matter was determined according to the International Dairy Federation (1987) method for milk and milk products. The protein content was determined by Kjeldahl on a Foss Kjeltec™ 8400 (Foss, Hillerød, Denmark). The procedure was modified from Koops et al. (1975) where a protein conversion factor of 6.38 was used in accordance with Merrill and Watt (1973).

### Chromatography

High performance liquid chromatography (HPLC) was carried out using a Waters 2695 separation module, a Waters 2487 dual wavelength absorbance detector running on Waters Empower® software (Milford, MA). Size-exclusion chromatography (SEC) was carried out on a TSK Gel G2000SW, 7.8 × 600 mm, column (TosoHaas Bioscience GmbH, Stuttgart, Germany) using an isocratic gradient of 30% MeCN containing 0.1% trifluoroacetic acid (v/v) at a flow-rate of 0.5 mL min^−1^ over 60 min. Samples of Alpha lactoalbumin (*α*-la), Beta lactoglobulin (*β*-lg) A and B, bovine serum albumin (BSA), Lactoferrin, and Caseinomacropeptide (CMP) (Sigma-Aldrich, Dublin, Ireland) were used as protein standards. Ribonuclease A, Cytochrome C, Aprotinin, Bacitracin, His-Pro-Arg-Trp, Leu-Trp-Met-Arg, Bradykinin, Leu-Phe, and Tyr-Glu (Bachem AG, Bubendorf, Switzerland) were used as molecular weight (*M*_w_) standards. All chromatography test samples and standards were made up in Milli-Q water (2.5 g L^−1^ solutions) prefiltered through 0.2 *μ*m low protein-binding membrane filters (Sartorius Stedim Biotech, GmbH, Goettingen, Germany) and 20 *μ*L applied to the column. The column elute was monitored at 214 nm and 280 nm and all solvents were filtered under vacuum through 0.45 *μ*m high velocity filters (Millipore Ltd., Durham, UK).

### In vitro Caco-2 cell culture

The human colonic adenocarcinoma cell line, Caco-2 (American Type Culture Collection, Manassas, VA), was maintained in Dulbecco's modified Eagle's medium (DMEM) (Invitrogen Corp., San Diego, CA) with 10% (v/v) fetal bovine serum (Invitrogen Corp.), 1% sodium pyruvate, 1% nonessential amino acids, 1% penicillin-streptomycin (Sigma-Aldrich Corp., St. Louis, MO) at 37°C in a humidified 5% CO_2_ incubator. The media was changed every alternative day and the cells were used for 21 days after it reached 99% confluence.

### Dose-dependent anti-inflammatory activity of casein hydrolysates

Caco-2 cells were maintained for 21 days before use in experiments in the conditions mentioned above. After 21 days, the Caco-2 cells were treated with TNF*α* (10 nmol/L) to stimulate a pro-inflammatory response. These TNF*α*-treated Caco-2 cells were simultaneously co-treated with casein hydrolysates or fractions at an increasing concentration of 0.01, 0.02, 0.05, 0.1, 0.5, 1, 2.5, and 5 mg/mL to optimize the concentration for the highest anti-inflammatory activity. The Caco-2 cells stimulated with TNF*α* and co-treated with the casein hydrolysates were incubated at 37°C for 24 h in a humidified 5% CO_2_ incubator. After 24 h, the media was collected and IL-8 concentrations were measured using Human CXCL8/IL-8 enzyme-linked immunosorbent assay (ELISA) kit (R&D Systems Europe, Ltd. Abingdon, UK) following the manufacturers protocol.

### Comparison of the anti-inflammatory activity of casein hydrolysates

Caco-2 cells were challenged with TNF*α* (10 nmol/L) (Sigma-Aldrich Corp.) to stimulate a pro-inflammatory response (control). Caco-2 cells challenged with TNF*α* and simultaneously co-treated with the commercially available anti-inflammatory steroid, Dexamethasone (10 nmol/L) (Sigma-Aldrich Corp.), was used as a positive control for this experiment. Similarly, Caco-2 cells were challenged with TNF*α* and co-treated with 1 mg/mL of NaCAS or EH or 5 kDaR or 1 kDaR or 1 kDaP to evaluate their anti-inflammatory property. The cell culture plates, after stimulating with TNF*α* and co-treating with/without either dexamethasone or NaCAS hydrolysates and fractions, were incubated at 37°C for 24 h in a humidified 5% CO_2_ incubator. After 24 h, the media was collected and IL-8 levels were measured using Human CXCL8/IL-8 ELISA kit, as mentioned in the above section.

### Ex vivo challenge to colonic tissues

Colonic tissues from three pigs were dissected along the mesentery and rinsed with sterile phosphate buffer saline (PBS). Tissue sections of 1 cm^3^ were stripped of overlying smooth muscle and placed in 1 mL of DMEM. Tissue explants from each animal was: (1) incubated in the presence of bacterial LPS, (Sigma Aldrich Corp.) at a concentration of 10 *μ*g/mL (challenged tissue); (2) in the presence of LPS (10 *μ*g/mL) and casein hydrolysate and fractions (1 mg/mL); and (3) in sterile DMEM (unchallenged tissue). All tissue explants were incubated at 37°C for 90 min before being removed, blotted dried, weighed and stored in 15 mL of RNAlater® (Applied Biosystems, Foster City, CA) overnight at 4°C. The RNAlater® was then removed prior to storing the samples at −80°C.

### RNA extraction

Total RNA was extracted using GenElute™ Mammalian Total RNA Miniprep Kit (Sigma-Aldrich Corp.) according to the manufacturer's instructions. Total RNA was subjected to DNAse I (Sigma-Aldrich Corp.) treatment, followed by further purification using a phenol-chloroform extraction method. The total RNA was quantified and assessed for purity using the NanoDrop®-ND1000 Spectrophotometer (Thermo Fisher Scientific Inc., Waltham, MA). The quality of the total RNA was determined by visualising on an ethidium bromide-stained 1% agarose gel.

### cDNA synthesis

Total RNA (1 *μ*g) was used for the synthesis of first strand cDNA using the First Strand cDNA Synthesis Kit (Qiagen Ltd. Crawley, UK) and oligo dT primers according to the manufactures instructions. The final volume of cDNA was adjusted to 120 *μ*L with nuclease free water.

### Quantitative real-time PCR

qPCR was carried out to quantify the following targets; interleukins (*IL1-α*,*IL1-β*,*IL-4*,*IL-6*,*IL-8*,*IL-10*,*IL-17*,*IL-21*), interferon (*IFN-γ*), tumor necrosis factor (*TNFα*), transforming growth factor (*TGF-β*) and forkhead box P3 (*FOXP3*). Primers used for the above mentioned targets are presented in Table1. The primer efficiency was determined using a serial dilution (1:4 dilution series over 7 points) of a cDNA pool, prepared by pooling an equal quantity of cDNA from all of the experimental samples, the efficiency of all primers was shown to be between 90 and 110%. Glyceraldehyde 3-phosphate dehydrogenase (*GAPDH*),*β*_2_ microglobulin (*B2M*), Beta-actin (*ACTB*), Peptidylprolyl isomerase A (*PPIA*), and 14-3-3 protein zeta/delta (*YWHAZ*) were used as endogenous controls as described by Ryan et al. (2010). All primers were designed using Primer Express™ software and were synthesized by MWG Biotech (Milton Keynes, UK). This assay was carried out using 96 well fast optical plates on a 7500HT ABI Prism® Sequence Detection System (PE Applied Biosystems, Foster City, CA) using Fast SYBR® Green PCR Master Mix (Applied Biosystems). All reactions were performed in triplicate in a total volume of 20 *μ*L containing 10 *μ*L Fast SYBR® PCR Master mix, forward and reverse primer (5 *μ*mol/L) (1 *μ*L), 8 *μ*L diethylpyrocarbonate-treated water and 1 *μ*L of template cDNA. The thermal cycling conditions were as follows, 95°C for 10 min, 40 cycles of 95°C for 15 sec and 65°C for 1 min. Dissociation analysis confirmed the specificity of the resulting PCR products.

**Table 1:** Oligonucleotide sequences of forward and reverse primers used in qPCR.

	Accession number	Forward primer (5′–3′)	*T*_m_ (°C)	Reverse primer (5′–3′)	*T*_m_ (°C)	Product length (bp)	Efficiency (%)
**Reference genes**							
*ACTB*	XM_001928093.1	GCACGGCATCATCACCAA	52.75	CCGGAGCTCGTTGTAGAAGGT	55.99	70	95.02
*PPIA*	NM_214353.1	CGGGTCCTGGCATCTTGT	62.1	TGGCAGTGCAAATGAAAAACT	60.7	75	100.26
*GAPDH*	AF017079.1	CAGCAATGCCTCCTGTACCA	62.2	ACGATGCCGAAGTTGTCATG	62.1	72	104.15
**Cytokine genes**							
*IL-1α*	NM_214029.1	CAGCCAACGGGAAGATTCTG	63.0	ATGGCTTCCAGGTCGTCAT	60.49	76	106.6
*IL-1β*	NM_001005149.1	TTGAATTCGAGTCTGCCCTGT	60.59	CCCAGGAAGACGGGCTTT	60.94	76	104
*IL-4*	HQ236500.1	CCAACCCTGGTCTGCTTACTG	61.8	TTGTAAGGTGATGTCGCACTTGT	58.9	71	95
*IL-6*	AB194100	AGACAAAGCCACCACCCCTAA	55.27	CTCGTTCTGTGACTGCAGCTTATC	59.92	69	99.99
*IL-8*	NM_213867.1	TGCACTTACTCTTGCCAGAACTG	61.9	CAAACTGGCTGTTGCCTTCTT	61.7	82	95.7
*IL-10*	NM_214041.1	GCCTTCGGCCCAGTGAA	63.4	AGAGACCCGGTCAGCAACAA	63.1	71	95.7
*IL-17A*	NM_001005729.1	CCCTGTCACTGCTGCTTCTG	60.57	TCATGATTCCCGCCTTCAC	60.40	57	101.2
*IL-21*	NM_214415	GGCACAGTGGCCCATAAATC	57.38	GCAGCAATTCAGGGTCCAAG	61.51	124	110
*IFN-γ*	NM_213948.1	TCTAACCTAAGAAAGCGGAAGAGAA	61.12	TTGCAGGCAGGATGACAATTA	61.54	81	94.4
*FOXP3*	NM_001128438.1	GTGGTGCAGTCTCTGGAACAAC	60.57	AGGTGGGCCTGCATAGCA	61.18	68	94
*TNF-α*	NM_214022.1	TGGCCCCTTGAGCATCA	62.5	CGGGCTTATCTGAGGTTTGAGA	62.8	68	91.5
*TGF β*	NM_214015.1	AGGGCTACCATGCCAATTTCT	60.63	CGGGTTGTGCTGGTTGTACA	61.68	101	93

### Normalization of data

Mean*C*_*t*_ values were converted into relative quantities using the formula: Relative quantity = (PCR efficiency)^Δ*Ct*^, where Δ*C*_*t*_ is the change in the*C*_*t*_ values of the sample relative to the highest expression (minimum*C*_*t*_ value). Relative quantities for the endogenous controls were input into geNorm (Vandesompele et al. 2002), a normalization factor was obtained from the four most stable (*M* < 1.5) reference genes (*GAPDH*,*B2M*,*ACTB* and*PPIA*) for colon. The relative quantities for the target genes were then divided by the normalization factor to give the final normalized value for each target gene in each sample.

### Statistical analysis

The current study was a complete randomized design experiment and data analyzed using the general linear model procedure of the Statistical Analysis Institute (SAS 2004). All the data were checked initially for normality using the PROC univariate procedure in SAS (2004). The values from the treatment groups were compared to unchallenged group using contrast statements. Probability values of <0.05 were used as the criterion for statistical significance. All results are presented in the tables as least square means ± standard error of the means (SEM).

## Results

### Compositional analysis and molecular weight distribution

The compositional analysis profile of NaCAS, EH of NaCAS and its associated fractions are presented in Table2. The 0.14 *μ*m fraction of EH did not have any significant activity and is not presented in the results. The pilot scale spray drying process resulted in a lower moisture content in the EH, 5 kDaR, 1 kDaR and 1 kDaP samples relative to the commercial NaCAS powder (*P < *0.01). The addition of NaOH during the hydrolysis process increased the ash content in the EH, 5 kDaR, 1 kDaR and 1 kDaP fractions relative to the NaCAS substrate (*P < *0.001). The hydrolysis process resulted in increased ash levels and decreased the lipid content in the NaCAS substrate, that is, 0.80 ± 0.06 g/100 g (NaCAS) to 0.62 ± 0.01 g/100 g (EH) (*P < *0.05). No lipids were detected in the size-fractionated products due to the use of the 0.14 *μ*m MF membrane. The protein content was analyzed by the Kjeldahl method and all samples had a protein content in the range of 84–90 g/100 g.

**Table 2:** Compositional characteristics of sodium caseinate (NaCAS), NaCAS enzyme hydrolysate and its retentates and permeate protein powders.

Test sample	Moisture (%)	Ash (g/100 g)	Lipid (g/100 g)	Protein^1^(g/100 g)
NaCAS	3.11 ± 0.09	3.90 ± 0.10	0.80 ± 0.06	88.66 ± 0.10
EH	1.87 ± 0.18**	5.41 ± 0.01***	0.62 ± 0.01*	89.38 ± 0.08**
5 kDaR	2.86 ± 0.39	6.80 ± 0.12***	ND	87.97 ± 0.91
1 kDaR	1.47 ± 0.26**	9.20 ± 0.07***	ND	86.13 ± 1.63
1 kDaP	2.00 ± 0.10**	9.40 ± 0.04***	ND	84.19 ± 1.25*

ND, not detected.

1 Kjeldahl conversion factor used was 6.38.

* *P* < 0.05,

** *P* < 0.01,

*** *P* < 0.001.

The molecular weight range of the NaCAS, EH of NaCAS and its associated fractions is presented in Table3. The majority of the NaCAS substrate had a molecular weight >30 kDa. The enzymatic hydrolysis of NaCAS decreased the abundance of material >10 kDa and increased the abundance of material <5 kDa (*P < *0.001). Whereas, the size fractionation increased the abundance of the material in their particular size range of 5–1 kDa and <1 kDa and >10 kDa protein fractions were not detected. The technique used in this process enriched the fractions of their respective size rather than accurately separating the proteins and the peptides according to size (Table3).

**Table 3:** Molecular weight distribution of sodium caseinate (NaCAS), NaCAS enzyme hydrolysate and its retentates and permeate protein powders.

Test sample	Molecular weight distribution (%)^1^				
	>30 kDa	30–10 kDa	10–5 kDa	5–1 kDa	<1 kDa
NaCAS	83.59 ± 0.22	14.55 ± 0.01	1.20 ± 0.01	0.61 ± 0.02	0.06 ± 0.01
EH	1.90 ± 0.01*	1.28 ± 0.01*	3.33 ± 0.06*	46.17 ± 0.01*	47.33 ± 0.01*
5 kDaR	0.09 ± 0.01*	0.31 ± 0.01*	1.93 ± 0.12*	56.25 ± 0.02*	41.52 ± 0.01*
1 kDaR	ND	0.06 ± 0.01*	0.36 ± 0.01*	28.67 ± 0.01*	70.90 ± 0.10*
1 kDaP	ND	ND	0.12 ± 0.01*	22.23 ± 0.03*	77.62 ± 0.04*

ND, not detected.

1 Molecular weight distribution was determined by SEC (TSK G2000SW) where the powders were reconstituted in distilled H_2_O to 2.5 g L^−1^ protein and subsequently filtered through a 0.45 *μ*m filter prior to the application of 20 *μ*L of this solution to the column.

* *P* < 0.01.

### Dose-dependent response and optimization of working concentration of casein hydrolysates and associated fractions

**Figure 1:**
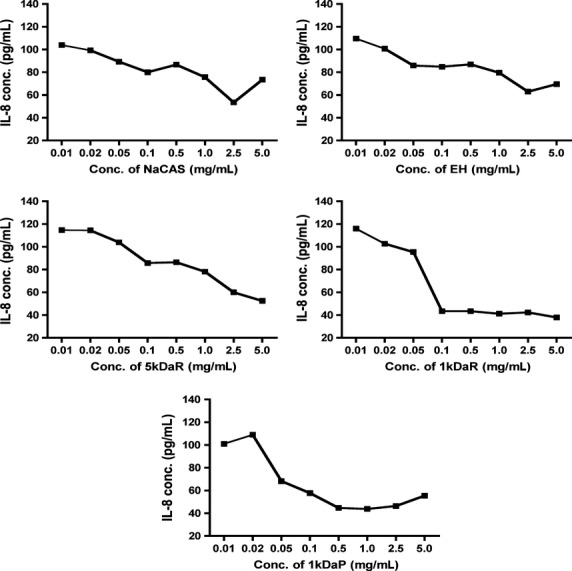
Dose dependent reduction of IL-8 concentration by a milk hydrolysate series (NaCAS, EH, 5 kDaR, 1 kDaR and 1 kDaP). Each data point indicates*n* = 3 ± standard error. The fully differentiated Caco-2 cells were challenged with*TNF*α and co-treated with milk hydrolysates for 24 h, cell lysate collected after 24 h and ELISA performed.

The dose-dependent anti-inflammatory response of the casein hydrolysate and associated fractions and the optimum working concentrations are presented in Figure1. All the samples from this series were associated with a dose-dependent anti-inflammatory response based on IL-8 concentrations. The co-treatment of TNF*α-*stimulated Caco-2 cells with concentrations of 1 mg/mL and above was consistently associated with a substantial reduction in IL-8 concentration over the 24 h period.

All the samples in this series had a consistent maximum anti-inflammatory effect at a concentration of 1 mg/mL evidenced by the reduction in IL-8 concentration in TNF*α-*stimulated Caco-2 cells.

### Anti-inflammatory effects of NaCAS hydrolysate and fractions in TNFα-stimulated Caco-2 cells

All Caco-2 cells were treated with TNF*α*, which stimulated the cells to produce IL-8 at a concentration of 110 ± 6.60 pg/mL over a 24 h period (control). The effects of co-treatment with either dexamethasone, NaCAS, NaCAS EH, retentates or permeate on IL-8 concentration relative to TNF*α-*stimulated control are presented in Table4. Co-treatment with dexamethasone resulted in a 41.6% reduction in IL-8 concentration (*P < *0.05) relative to control. Similarly, co-treatment with NaCAS or EH or 5 kDaR resulted in a reduction of 31.1% (*P < *0.05), 31% (*P < *0.05) and 32.7% (*P < *0.05), respectively in IL-8 concentration relative to control. The greatest effect was observed following co-treatment with either the 1 kDaR or 1 kDaP, which resulted in the 68.7% (*P < *0.01) and 66.15 (*P < *0.01), respectively, reduction in IL-8 concentration relative to control.

**Table 4:** Effect on*IL-8* production in fully differentiated Caco-2 cells stimulated with*TNFα* and co-treated with dexamethasone, sodium caseinate (NaCAS) and NaCAS enzyme hydrolysate, retentates, and permeate for 24 h.

Treatment	IL-8 conc. (pg/mL)	SEM	% reduction^1^	Significance
TNF*α*	110.0	6.60		
TNF*α* + Dexamethasone	68.4	5.87	41.6	*
TNF*α* + NaCAS	75.8	3.35	31.1	*
TNF*α* + EH	79.0	5.11	31.0	*
TNF*α* + 5 kDaR	77.3	4.44	32.7	*
TNF*α* + 1 kDaR	41.3	8.00	68.7	**
TNF*α* + 1 kDaP	43.9	7.82	66.1	**

Cell lysate was collected after 24 h of treatment and ELISA was performed.

1 The % reduction values are relative to control (TNF*α* stimulated) Caco-2 cells.

* *P* < 0.05,

** *P* < 0.01,*n* = 3.

### Anti-inflammatory effects of NaCAS hydrolysates in LPS-stimulated porcine colonic tissue explants

#### Effect of LPS on porcine colonic tissue explants

**Figure 2:**
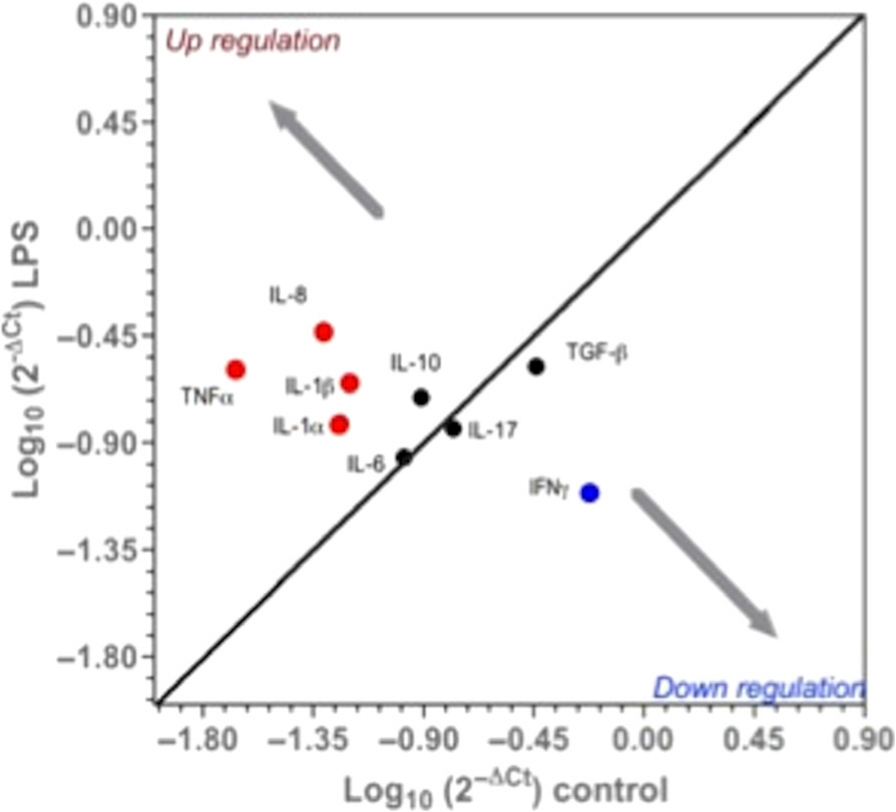
Difference in expression of a selected panel of cytokine genes between unchallenged and Lipopolysaccharides (LPS)-challenged ex vivo colonic tissues. The red dots represent significant upregulated genes (*P < *0.05), the blue dot represents significant downregulated gene (*P < *0.05) and black dots represent genes not affected by LPS (*P* > 0.05).

The effect of LPS challenge on the expression of a selected panel of cytokine genes from ex vivo colonic tissues is presented in Figure2. The LPS-challenged porcine colonic tissues were associated with increases in the normalized relative quantities of*IL1-α* (0.424 vs. 0.064 ± 0.091,*P < *0.05)*, IL1-β* (0.442 vs. 0.062 ± 0.102,*P < *0.01)*, IL-8* (0.212 vs. 0.064 ± 0.055,*P < *0.05) and*TNFα* (0.234 vs. 0.024 ± 0.037,*P < *0.001) compared to unchallenged colonic tissues. LPS-challenged tissues were also associated with a decrease in*IFN-γ* expression relative to unchallenged tissues (0.082 vs. 0.644 ± 0.047,*P < *0.001). LPS had no effect on*IL-6*,*IL-10*,*IL-17* and*TGF-β* expression, while*IL-4*,*IL-21* and*FOXP3* expression was nondetectable in the colonic tissues used in this study.

#### Effect of NaCAS hydrolysates on LPS-stimulated porcine colonic explants

**Figure 3:**
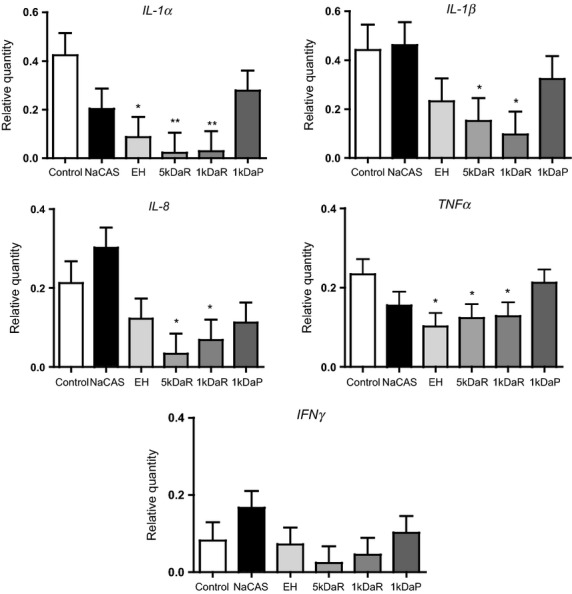
Effect of NaCAS, EH, 5 kDaR, 1 kDaR or 1 kDaP on relative quantity (RQ) of a selected panel of cytokine genes stimulated by LPS challenge for 3 h in porcine colonic tissue explant. After challenge with LPS and treatment with milk hydrolysates, total RNA was extracted, cDNA synthesised and RT-qPCR was performed to quantify the selected panel of genes. RQ values of treatments with NaCAS, EH, 5 kDaR, 1 kDaR or 1 kDaP compared to LPS challenged control, error bars indicate SE. *P < 0.05, **P < 0.01.

The effect of NaCAS, NaCAS EH, retentates, and permeates on gene expression in the LPS-stimulated tissue is presented in Figure3. Co-treatment of colonic tissues with hydrolysates EH, 5 kDaR and 1 kDaR was associated with a downregulation of*IL1-α* (0.086, 0.021, 0.028 vs. 0.424 ± 0.083,*P < *0.05) and*TNFα* (0.101, 0.123, 0.128 vs. 0.234 ± 0.034,*P < *0.05) expression relative to LPS-challenged tissues. Similarly, co-treatment with 5 kDaR and 1 kDaR hydrolysates was associated with a downregulation of*IL-1β* (0.151, 0.096 vs. 0.442 ± 0.093,*P < *0.05) and*IL-8* (0.033, 0.068 vs. 0.212 ± 0.050,*P < *0.05) expression relative to LPS-challenged tissues.

**Figure 4:**
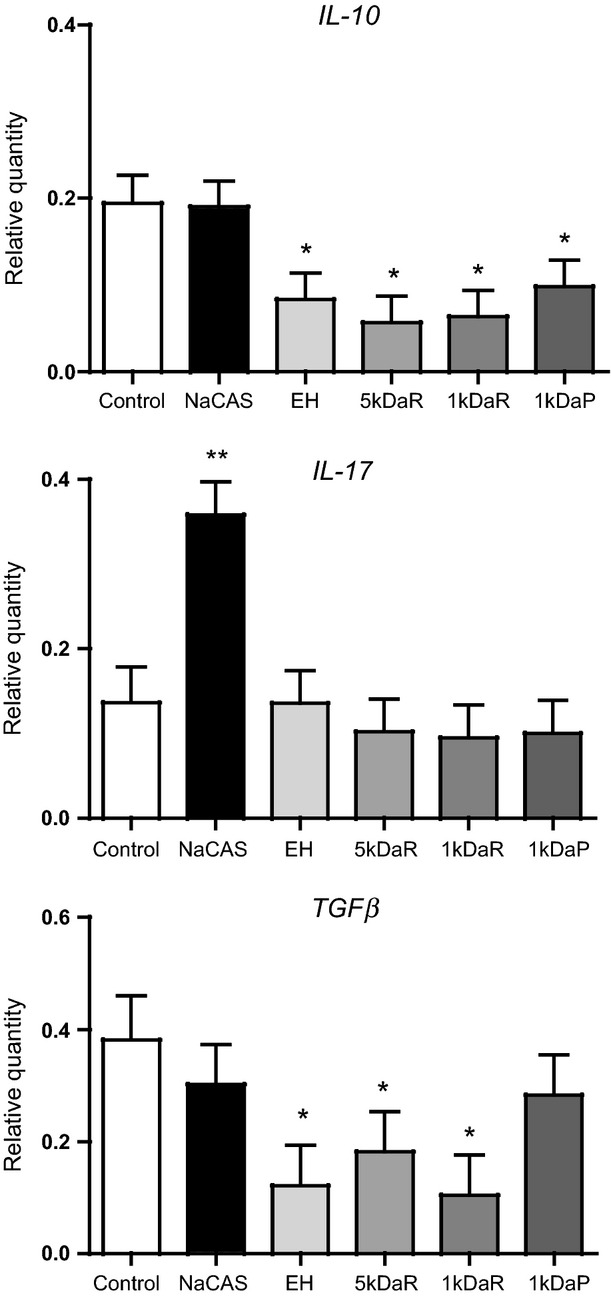
Effect of NaCAS and NaCAS enzyme hydrolysate, retentates and permeate on relative quantity (RQ) of genes un-stimulated by LPS challenge for 3 h in porcine colonic tissue explant. After 3 h of treatment with milk hydrolysates, total RNA was extracted, cDNA synthesised and RT-qPCR performed to quantify the selected panel of genes. RQ values of treatments with NaCAS, EH, 5 kDaR, 1 kDaR or 1 kDaP compared to unchallenged control, error bars indicate SE. *P < 0.05, **P < 0.01.

While the LPS challenge had no effect on the expression of*IL-10*,*IL-17* and*TGF-β,* as presented in Figure2, co-treatment with casein hydrolysates had significant effects on*IL-10* and*TGF-β* expression, whereas co-treatment with NaCAS had significant effects on only*IL-17* expression relative to unchallenged tissues, presented in Figure4. The treatment of porcine colonic tissues with EH, 5 kDaR, 1 kDaR and 1 kDaP caused a down-regulation of*IL-10* expression (0.083, 0.056, 0.063, 0.098 vs. 0.194 ± 0.029,*P < *0.05) relative to unchallenged tissues. Colonic tissues co-treated with NaCAS was associated with an increase in*IL-17* (0.358 vs. 0.136 ± 0.042,*P < *0.05) expression relative to unchallenged tissues. The co-treatment with EH, 5 kDaR and 1 kDaR hydrolysates was associated with a down-regulation of*TGF-β* (0.121, 0.181, 0.105 vs. 0.382 ± 0.071,*P < *0.05) expression in colonic tissues relative to unchallenged tissues.

## Discussion

IL-8 is a chemotactic cytokine and an established marker for inflammation in the gastrointestinal system (Harada et al. 1994; Mahida 2000). Increased serum IL-8 has been associated with chronic inflammatory diseases including IBD, and is a potential target for immunotherapeutic approaches (Skov et al. 2008). The ability of the NaCAS hydrolysates to suppress IL-8 in the TNF*α-*stimulated Caco-2 cell system was an initial strong indicator of the anti-inflammatory activity of these hydrolysates, with both the 1 kDaR and 1 kDaP suppressing IL-8 secretion relative to NaCAS, NaCAS EH, 5 kDaR, and dexamenthasone. This observation was further supported by the fact that the hydrolysate fractions, in particular the 5 kDaR and 1 kDaR maintained their anti-inflammatory properties in the ex vivo colonic system, reducing the expression of a panel of pro-inflammatory cytokines*IL1-α*,*IL1-β*,*IL-8*,*IL-10*,*TNFα* and*TGF-β* in LPS-challenged porcine colonic tissues. The fact that the expression of all of these cytokines play a role in the progression of inflammatory conditions such as IBD raise the possibility that the hydrolysate fractions identified in this study may have the potential to alleviate the recurrence of IBD-like conditions.

The functional similarity of Caco-2 cells to colonic enterocytes, along with their ability to elicit a pro-inflammatory reaction in response to stimulants like H_2_O_2_, LPS and TNF*α* makes them a useful in vitro model for the study of effects of food ingredients on the gut epithelial layer (Tanoue et al. 2008). In this study and in the previous study by Piccolomini et al. (2012), modified milk proteins have been shown to suppress IL-8 concentrations in cells already exhibiting inflammation. The anti-inflammatory effects of fractions in this study indicate that the bioactive molecules within these fractions could be useful agents in the re-establishment of GIT homeostasis, which may have been lost due to disregulated inflammation. While all the casein hydrolysates induced a reduction in IL-8 production, the greatest anti-inflammatory activity was observed in response to exposure to the 1 kDaR and 1 kDaP fractions. This greater anti-inflammatory activity is most likely attributable to the <1-kDa protein fractions, which are enriched in these two fractions.

The anti-inflammatory properties of casein hydrolysates were subsequently evaluated in an ex vivo system using porcine colonic tissue explant system. The ex vivo system has several advantages over the in vitro Caco-2 cell system. Firstly, porcine colonic tissue ex vivo closely represents the in vivo complexity of colonic tissue, which is composed of heterogeneous cell types. Secondly, ex vivo colonic tissue response to inflammation inducing agents such as LPS and this response mimics a microbial challenge within the gut. LPS acts via the TLR4/MD-2 complex and can stimulate a number of pro-inflammatory cytokines in the tissue explants (Moue et al. 2008; Yuk and Jo 2011) as some of these signaling factors would otherwise have negligible expression levels. In the present study, ex vivo treatment of porcine colonic tissues with LPS resulted in an increase in the expression of the pro-inflammatory cytokine genes:*IL1-α*,*IL1-β*,*IL-8*, and*TNFα*. In the gut of a live animal/human, these pro-inflammatory cytokines are generally secreted by macrophages present under the mucosal layer of the intestine (Mahida 2000). The ex vivo results from this study indicate that the 1 kDaR reduced the expression of cytokines*IL1-α*,*IL1-β*,*IL-8*,*IL-10*,*TNFα*, and*TGF-β* in porcine colonic tissues. Increased expression of the pro-inflammatory cytokines has been associated with a number of metabolic syndromes (Cam and de Mejia 2012) in addition to chronic inflammation of the bowel in humans (Skov et al. 2008). In line with the Caco-2 cell system study, both the 5 kDaR and 1 kDaR hydrolysates downregulated*IL-8* gene expression in colonic explants. IL-8 is produced in the intestinal mucosa by epithelial cells, macrophages, and fibroblasts (Daig et al. 1996). Human subjects exhibiting clinical signs of ulcerative colitis (UC) were shown to have decreased levels of*IL-8* (upon receiving the partition-herb moxibustion) in their mucosae which was coincident with improvements in the histological profile of the colon (Zhou et al. 2009). Hence, casein hydrolysates could have potential as therapeutic agents in the treatment of inflammatory diseases of the GIT that are linked to an excessive activity of IL-8, for example, UC. The EH, 5 kDaR and 1 kDaR caused a down-regulation of*IL1-α* expression, a cytokine which mediates immune and inflammatory responses and is generally produced by macrophages, monocytes, neutrophils, and endothelial cells (Dinarello 1994). Two casein hydrolysate fractions (5 kDaR and 1 kDaR) also downregulated the*IL1-β* gene expression in this study.*IL-1β* is also a member of*IL-1* family and plays an important role in the Th17-mediated immune response (Chung et al. 2009). In this study,*TNFα* gene expression was also downregulated in response to EH, 5 kDaR and 1 kDaR.*TNFα*, is a key player in inflammation implicated in the progression of IBD (Skov et al. 2008) Serum levels of*TNFα* produced from macrophages or monocytes is correlated with the clinical progression of IBD, further supporting the use of anti-TNF*α* antibodies in IBD therapy (Chaparro et al. 2012). Hence, the downregulation of both of these cytokines provides valuable evidence of the anti-inflammatory properties of the 5 kDaR and 1 kDaR.

In disease conditions such as IBD, progression of T-cell differentiation and inhibition depends upon a number of cytokines including, IL-4, IFN-*γ*, and TGF-*β* (Zhu and Paul 2008). Although in this study, the expression of*IL-4* and*IFN-γ* genes was unaffected by milk hydrolysates,*TGF-β* expression was altered.*TGF-β* regulates growth, differentiation and function of immune cells by inhibiting T cells and switching on B cells (Maloy and Powrie 2011). Greater expression of*TGF-β* has been reported in the intestines of IBD patients (Franke et al. 2008) and its excessive production has been linked to increased fibrosis, stimulation of collagen production, and increased incidences of carcinogenesis (Sebens and Schäfer 2012).

Our study indicated that EH and all the size fractions of casein hydrolysate downregulated*IL-10* expression in porcine colonic explant. A genome-wide association study identified*IL-10* as a susceptibility locus for the development of IBD in humans (Franke et al. 2008). It has also been reported that local levels of*IL-10* mRNA were higher in mucosa of IBD patients (Niessner and Volk 2008). Recent reports have indicated that Treg cells play a role in the regulation of IL-10 production. IL-10 is a factor, which generally suppresses inflammatory immune responses and has been shown to be important in the maintenance of immune homeostasis within the gut (Tanoue and Honda 2012). In situ analysis of healthy human colonic mucosa using qPCR and immunoblot analysis has indicated that*IL-10* functions as an immunomodulatory cytokine and possesses both immunosupressive and pro-inflammatory functions depending on the context (Jarry et al. 2008).*IL-10* regulates immune function through T cells, B cells, dendritic cells, and intestinal epithelial cells. This cytokine also plays an important role in the inhibition of the Th1 cells as well as diminishing the antigen presenting capacity of monocytes (Kaser et al. 2010).

The potential of the NaCAS hydrosylate fractions to suppress the inflammatory cytokines has particular relevance to inflammatory condition of the GIT including IBD. IBD represents a group of painful, debilitating disease conditions of the lower gastrointestinal tract that affects an estimated 2.4 million people in Europe (Cosnes et al. 2011). While IBD is known to be a problem for adults, recent analyses highlight that fact that the incidence of IBD is also increasing in children (Langholz 2010; Malaty et al. 2010). A characteristic of IBD is an exaggerated immune response to enteric microorganisms, with a resultant overproduction of pro-inflammatory cytokines and chemokines. These signalling molecules induce inappropriate recruitment and infiltration of lymphocytes and granulocytes to the colonic epithelial mucosa eliciting localized inflammation (Kim et al. 2010). While therapeutics are currently available for IBD, a key feature of the disease is a tendency for relapse, and a recurrent pattern of inflammation (Malaty et al. 2010). The identification of natural modulatory bioactives, such as the NaCAS hydrosylate fractions identified in this study, that can be taken in the diet which can retain inflammatory homeostasis would be of great benefit in decreasing the incidence of IBD recurrence, and warrants further research.

## Conclusion

The study demonstrates the consistent anti-inflammatory bioactivity of a 1-kDaR of NaCAS hydrolysate in both in vitro and ex vivo colon models. The 1-kDaR was associated with a reduced IL-8 concentration in vitro and down regulation of a panel of pro-inflammatory cytokines in the ex vivo colonic explant system. These findings indicate that further analysis of this 1-kDaR of NaCAS hydrolysate would be valuable in the exploration of its potential as an anti-inflammatory functional food ingredient.
